# Creative Solutions: An Art-Based Intervention to Address Burnout in CAMHS

**DOI:** 10.1192/bjo.2025.10444

**Published:** 2025-06-20

**Authors:** Amelia Smith, Erica Pietrogrande

**Affiliations:** North London NHS Foundation Trust, London, United Kingdom

## Abstract

**Aims:** Workplace stress and burnout are common within healthcare. The NHS Staff Survey 2023 found that approximately one third of respondents were experiencing burnout and were considering leaving the organisation. Studies suggest this is significantly higher in Child and Adolescent Mental Health Services (CAMHS). The negative impact of burnout on staff wellbeing, retainment, and patient care is well-recognised. Arts-based interventions have been shown to improve work-related stress and promote empathy and resilience. We set up a regular reflective art group for staff at an adolescent mental health team to see if this would reduce stress and improve wellbeing and job satisfaction.

**Methods:** We set up a monthly recurrent one-hour group, attended by the whole multidisciplinary team at the Service for Adolescents and their Families in Enfield (SAFE) and facilitated by team members with experience of art therapy. Group attendees were encouraged to express themselves in visual artwork freely, without specific instructions.

We anonymously surveyed attendees at baseline and at two- and four-months following implementation of the group to measure general and workplace-specific wellbeing, stress and burnout, job satisfaction, and staff attitudes to the intervention. We collected both quantitative and qualitative data.

**Results:** Out of a team of 19 members, 10 on average attended each session over 5 months. All disciplines and seniority levels were represented.

We collected 12 responses in March 2024 before implementing the group, 7 responses in May 2024 and 6 responses in July 2024 (2 and 4 months after implementation respectively).

The group was rated 5 out of 5 stars unanimously and described as a “space to connect with colleagues, reflect, and calm stress and emotional impact of work”.

Overall, there was an improvement in team wellbeing, job satisfaction and confidence from March to July, despite an increase in perceived stress and a decrease in general mental wellbeing. The findings can be better understood within the wider context of the service at the time, namely worsening staff shortages and extreme workload pressures.

**Conclusion:** The art group was very well received by colleagues and appeared to boost morale during a particularly difficult period for the team. However, this alone was not sufficient to reduce the stress derived from trying to meet clinical demands with inadequate staffing levels.

Interventions improving job satisfaction, like this project, could play an important role in fighting workforce erosion if combined with long-term commitment to a sustainable workforce on an institutional level.

